# Biomechanical Investigation of Lower Limbs during Slope Transformation Running with Different Longitudinal Bending Stiffness Shoes

**DOI:** 10.3390/s24123902

**Published:** 2024-06-16

**Authors:** Runhan Lu, Hairong Chen, Jialu Huang, Jingyi Ye, Lidong Gao, Qian Liu, Wenjing Quan, Yaodong Gu

**Affiliations:** 1 Faculty of Sports Science, Ningbo University, Ningbo 315211, China; lurunhan1@163.com (R.L.); hjialu@126.com (J.H.); yejingyi1999@gmail.com (J.Y.); liuqian19981008@163.com (Q.L.); 2 Doctoral School on Safety and Security Sciences, Óbuda University, 1034 Budapest, Hungary; chenhairong233@163.com; 3 Faculty of Engineering, University of Szeged, 6724 Szeged, Hungary; 4 Department of Material Science and Technology, Audi Hungaria Faculty of Automotive Engineering, Széchenyi István University, 9026 Győr, Hungary; gaolidong1997@hotmail.com

**Keywords:** level, uphill, running, kinematics, kinetics, longitudinal bending stiffness

## Abstract

Background: During city running or marathon races, shifts in level ground and up-and-down slopes are regularly encountered, resulting in changes in lower limb biomechanics. The longitudinal bending stiffness of the running shoe affects the running performance. Purpose: This research aimed to investigate the biomechanical changes in the lower limbs when transitioning from level ground to an uphill slope under different longitudinal bending stiffness (LBS) levels in running shoes. Methods: Fifteen male amateur runners were recruited and tested while wearing three different LBS running shoes. The participants were asked to pass the force platform with their right foot at a speed of 3.3 m/s ± 0.2. Kinematics data and GRFs were collected synchronously. Each participant completed and recorded ten successful experiments per pair of shoes. Results: The range of motion in the sagittal of the knee joint was reduced with the increase in the longitudinal bending stiffness. Positive work was increased in the sagittal plane of the ankle joint and reduced in the keen joint. The negative work of the knee joint increased in the sagittal plane. The positive work of the metatarsophalangeal joint in the sagittal plane increased. Conclusion: Transitioning from running on a level surface to running uphill, while wearing running shoes with high LBS, could lead to improved efficiency in lower limb function. However, the higher LBS of running shoes increases the energy absorption of the knee joint, potentially increasing the risk of knee injuries. Thus, amateurs should choose running shoes with optimal stiffness when running.

## 1. Introduction

Recently, running has gained significant popularity as an accessible and inclusive sport. Running performance derives from a combination of anatomical, physiological, and behavioral traits that are uniquely evolved in humans [1]. As a result, research on the physiology and biomechanics of running never stops. This research has the potential to significantly improve human running performance. Most studies have focused on level running; however, in a marathon or city run, the route includes not just level ground but also uphill and downhill sections. For example, the Comrades Marathon, a world-famous ultra-marathon that has been held in South Africa since 1921, is a 90-km marathon with a course that incorporates a variety of road conditions (switching between uphill, level, and downhill). There was research discovered that uphill running leads to a decrease in both the maximal capacity to store and release elastic energy, in comparison to running on level ground. As a result, the body needs to generate additional mechanical energy to make up for the reduced elastic energy storage [1]. Much of this additional mechanical energy is generated by the hip [2]. During uphill walking, the ankle joint needs to release more energy to propel the body forward, while the absorption of energy by the ankle remains essentially constant [3]. For the knee joint, the angle of activity was greater when running uphill than when running on level ground [4]. In addition, when running downhill, the negative work absorbed by the knee increases, thereby increasing the risk of injury. In uphill running, the hip joint releases more energy and has an increased range of motion [5]. When jogging uphill at a consistent pace, the joints focus on releasing energy rather than using it up. It is vital to research how to release more energy to increase a runner’s exercise performance.

The selection of appropriate running shoes is crucial for enhancing exercise performance and reducing the risk of sports-related injuries [6]. The longitudinal bending stiffness (LBS) of running shoes has been recognized as a key consideration in the development of performance footwear as part of the design to improve runner’s exercise performance as well as minimize sports injuries [7,8]. The longitudinal bending stiffness of running shoes reduces the runner’s walking frequency, prolongs the swing phase, and increases the peak vertical ground reaction and vertical impulse for each step [9]. Running economy is an important factor in determining long-distance running, and increasing the longitudinal bending stiffness of shoes could reduce the energy cost of running and effectively improve the running economy [10,11]. In addition, improving the LBS of a shoe has a significant impact on the ankle as well as the metatarsophalangeal joints. Properly fitted LBS running shoes play a key role in comfort as well as in the improvement of exercise performance [12]. Research has indicated that the metatarsophalangeal joints experience significant extension and make negative work while running, and this energy absorption has a negative impact on exercise performance. Increased LBS inhibits the extension of the metatarsophalangeal joint and reduces the time the supporting metatarsophalangeal joint goes from dorsiflexion to plantarflexion, thereby reducing the energy loss of the metatarsophalangeal joint during the running stance phase [13,14]. As Cigoja et al. found that increasing the LBS shortened the peak contraction velocity of the tendon unit of the calf muscles, these changes will result in a decrease in energy expenditure in the ankle plantarflexors and an increase in energy return to the Achilles tendon [15]. Additionally, an increase in LBS shifts the center of gravity of lower limb joint work from the knee to the metatarsophalangeal joints. Research has shown that as the LBS increases, there is a decrease in positive work at the knee joint and a significant increase in positive work at the metatarsophalangeal joints. This indicates a redistribution of work within the lower limb joints, with more work being redistributed from the knee to the ankle [16,17]. Furthermore, the increase in LBS results in a heightened rigidity of the shoe, providing enhanced support to the joints, exhibiting less mobility in the ankle and knee joints, and resulting in increased joint stability [18]. However, the transition from a level surface to an uphill slope in running shoes with different LBS has not been well studied. 

Therefore, the research aimed to examine the variations in lower limb biomechanics during the transition from level to uphill running while wearing different LBS running shoes. The knee, ankle, and metatarsophalangeal joints of amateur runners transitioning from level to uphill running were analyzed in different LBS running shoes, helping to identify the most acceptable stiffness for them.

## 2. Materials and Methods

### 2.1. Participants

The sample size was calculated using G*Power 3.1 (Franz Faul, Germany) for univariate analysis of variance for detecting the number of groups = 1, number of measurements = 3, and power = 0.8 [19]. Based on these parameters, it was estimated that a minimum of 15 participants would be required for this research. A total of 15 male amateur runners were recruited (shoe size: 41, age: 22.5 ± 1.43, height: 175.3 ± 1.64 cm, weight: 65.25 ± 1.59 kg). Amateur runners are defined as running at least 3 times a week for 45 min or 10 km [20]. The participants’ dominant leg is the right leg (the leg of the football goalkeeper). During the year prior to participation in the experiment, the subjects did not have severe lower limb injuries and surgeries, and before the experiment began, participants were asked to avoid any intense physical activity for 48 h to eliminate the possibility of fatigue. A healthy diet and full rest were required before the experiment. Alcohol or caffeine of any kind was also prohibited within 24 h of the start of the experiment [21]. All participants had to sign a written informed consent. The experiment was approved by the Ethics Committee of the University of Ningbo (2024RBG3272).

### 2.2. Experimental Processes

**Figure 1:**
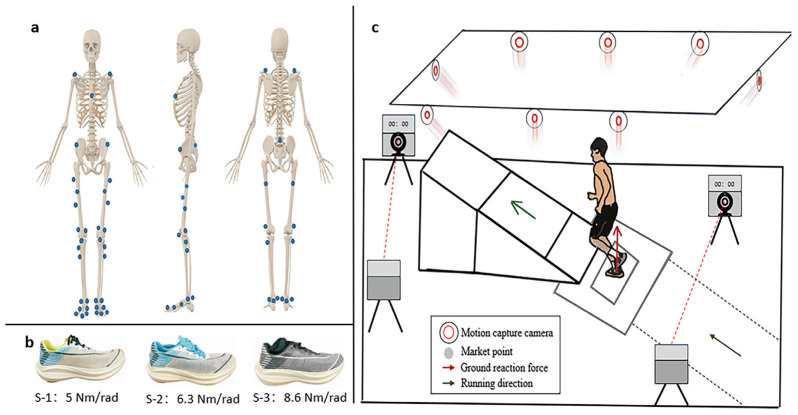
(**a**) The front, side, and back positions of markers. Blue dots: markers. (**b**) Three pairs of experimental shoes. (**c**) Display of experiment design for collecting the biomechanical data during the running stance phase.

Prior to the official start of the experiment, participants were asked to warm up by walking at 2.2 m/s for 1 min on the treadmill [22]. We uniformly provided the participants with clothing and shoes to avoid experimental differences and maintain consistency. In the official experiment, the participants were asked to apply 38 reflective markers (diameter: 14 mm) [23]. The specific points are shown in Figure 1a. The participants were asked to step with their right foot on the force platform with both eyes looking forward until the complete static coordinates were captured. During the test, the participants were wearing the supplied clothing as well as shoes. In this research, based on the longitudinal bending stiffness selected for shoes in previous research (S-1: 5.0 Nm/rad, S-2: 6.3 Nm/rad, S-3: 8.6 Nm/rad), except for the difference in longitudinal bending stiffness, the other mechanical properties of the three pairs of running shoes were the same [13,24,25]. The LBS values of the shoes were measured by a rotational axis material-testing machine (Instron ElectroPuls E1000, Norwood, MA, USA). Then the participants passed the slope at a speed of 3.3 m/s ± 0.2 m/s [26]. The shoes are shown in Figure 1b. The longitudinal bending stiffness of the running shoes tested in this study was modeled on Willwacher’s study, which was closer to previous studies and had not been tested in previous studies of transitioning from level to uphill running. The participants were asked to run with their right leg stepping on the force platform every time they switched from the plane to the uphill (Figure 1c). Each pair of shoes was tested 10 times and valid data were obtained. After each pair of shoes was tested, the participants rested for five minutes to prevent fatigue. The running data from the standing phase of an amateur runner were captured using the Vicon motion capture system (Vicon Metrics Ltd., Oxford, UK). Vertical ground reaction forces were used to identify the amateur runner’s toe contact to toe-off. To control experimental variables, we used a Brauer timing light (Brower Timing System, Draper, UT, USA) to control running speed.

### 2.3. Data Collection and Processing

This research analyzed the biomechanical properties of amateur runners’ limbs during the transition from running on a level surface to running uphill. We converted the C3D file data exported by Vicon into ‘.trc’ and ‘.mot’ files. Then it was imported into OpenSim for the next step [27]. A musculoskeletal model from the OpenSim website (Gait 2392) was used [28,29]. The models in OpenSim were scaled to utilize the participant’s marker point location and weights. Until the error value between the experimental and virtual markers was less than 0.02, the scaled model was applied to the data calculation. The inversion kinematics (IK) calculation tool was used in OpenSim to calculate the joint angle and optimize the results using the minimum binary method to minimize the error between experimental and virtual markings. Inversion Dynamics (ID) calculates the net moment of the knee, ankle, and thigh joints. The ID tool performs inversion dynamic analysis by applying the data of the kinematics of a given model description and the partial kinetics that may be applied to the model. The ID tool deals with the equations of force and acceleration in classical mechanics in the inversion dynamic sense, obtaining the net moment and torque of each joint that produces motion, from the data of IK and ID, the joint power and work performed are then calculated [30].

### 2.4. Statistical Analysis

The knee, ankle, metatarsophalangeal joint angles, peak moment, power, and work on the part of the plane were analyzed in SPSS 26.0 (IBM, Albany, NY, USA). The data obtained from the experiment used mean ± standard differential representation [31]. First, tests for normality and homogeneity of variances (Shapiro–Wilk and Levene’s, respectively) were conducted on all data before the analysis. A one-way repeated measure ANOVA was utilized to analyze the impact of running in running shoes with different LBS on lower limb biomechanics, in accordance with tests for normality and homogeneity of variance. The significance level was set to *p* < 0.05. Post hoc tests were compared using the Bonferroni method to determine which of the two LBS running shoe conditions had significant differences in the range of motion, peak moments, peak positive and negative power, and positive and negative work at the hip, knee, ankle, and metatarsophalangeal joints based on *p*-values. The *p*-values require 3 comparisons, and the probability of 0.05 is divided by the number of comparisons to be made, 3, so the level of significance is set at α = 0.017, and a difference of *p* < 0.017 is considered statistically significant.

## 3. Results

### 3.1. Kinematics

**Figure 2:**
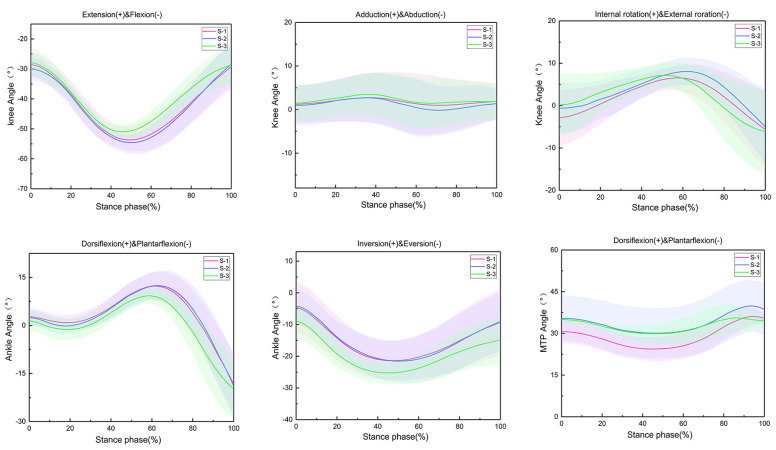
Changes in stance phase and knee, ankle, and metatarsophalangeal joint angles in three running shoe conditions.

The changes in knee, ankle, and metatarsophalangeal joint angles during the stance phase of running in amateur runners in three different LBS running shoe conditions are shown in Figure 2; the range of joint motion is shown in Table 1. In three different LBS running shoe conditions, significant differences were seen in the motion angle of the knee adduction/abduction, as well as the metatarsophalangeal joint plantarflexion/dorsiflexion. As the LBS increased, joint angles decreased. There was no significant difference in the motion angle of the different planes of the other joints.

### 3.2. Kinetics

**Figure 3:**
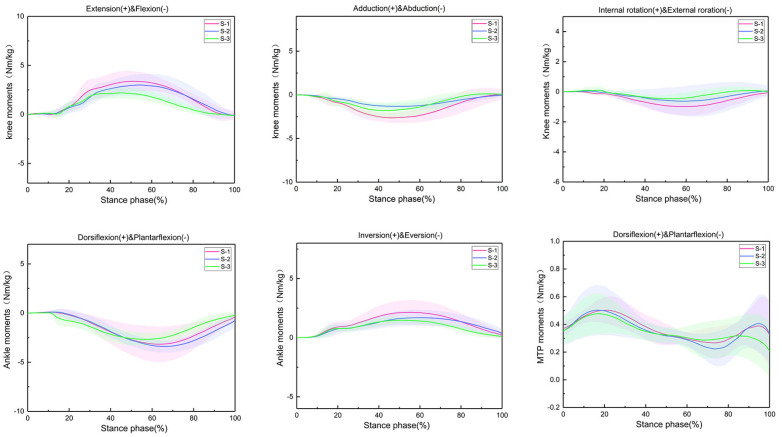
Changes in knee, ankle, and metatarsophalangeal joint moments during the support period in the three running shoe conditions.

The changes in the knee, ankle, and metatarsophalangeal joint peak moment during the stance phase of running in amateur runners in three different LBS running shoe conditions are shown in Figure 3; the peak joint moment is shown in Table 1. In three different LBS running shoe conditions, significant differences were seen in the peak moments of knee flexion/extension, adduction/abduction, and ankle inversion/eversion. As the LBS increased, the peak joint moment decreased. There was no significant difference in the peak moment of the different surfaces of the other joints.

The changes in knee, ankle, and metatarsophalangeal joint peak positive and negative power during the stance phase of running in amateur runners in three different LBS running shoe conditions are shown in Table 1. In three different LBS running shoe conditions, significant differences were seen in the peak positive power of knee flexion/extension, adduction/abduction, and ankle plantarflexion/dorsiflexion, and significant differences were seen in the peak negative power of knee adduction/abduction as well as ankle plantarflexion/dorsiflexion. The peak positive and negative power of joints decreased with increasing LBS. There was no significant difference in the peak positive and negative power of the different surfaces of the other joints.

The changes in knee, ankle, and metatarsophalangeal joint positive and negative work during the stance phase of running in amateur runners in three different LBS running shoe conditions are shown in Table 1. In three different LBS running shoe conditions, significant differences were seen in the positive work of knee adduction/abduction, plantarflexion/dorsiflexion with the ankle and metatarsophalangeal joint, and inversion/eversion with the ankle, and significant differences were seen in the negative work of knee flexion/extension, internal-external, and ankle plantarflexion/dorsiflexion. As the LBS increased, the positive work of knee adduction/abduction and ankle inversion/eversion decreased, while the plantarflexion/dorsiflexion of the ankle and metatarsophalangeal joint increased, the negative work of knee flexion/extension and ankle plantarflexion/dorsiflexion increased, and the internal-external of the knee decreased. There was no significant difference in the positive and negative work of the different surfaces of the other joints.

## 4. Discussion

This research aims to investigate the kinematics and kinetics of the knee, ankle, and metatarsophalangeal joints in amateur runners transitioning from level to uphill running while wearing different LBS running shoes. When transitioning from level to uphill running, our research observed that, as the LBS increased, there was a decrease in the range of motion and positive work of the knee, an increase in the positive work of the ankle, and a decrease in the range of motion and negative work of the metatarsophalangeal joint, together with an increase in positive work.

From a kinematic point of view, there was a significant difference in the knee joint angle when transitioning from level to uphill running in different LBS running shoes, and the S-2 shoe showed a higher range of knee adduction/abduction compared to the S-3 shoe. Thorsten Sterzing’s research found that the characteristics of running shoes affect the knee and ankle range of motion during running, which impacts the control of knee and ankle stabilization [18]; a decreased knee and ankle range of motion indicates that higher LBS improves knee stability in amateur runners transitioning from level to uphill running. Moreover, the rise in LBS enhances the shoe’s stiffness, resulting in improved foot support. Shoes with low stiffness offer less foot support than shoes with high stiffness, leading to amateur runners having difficulty controlling the stability of the knee joint. Additionally, significant differences in the range of motion of the metatarsophalangeal joints were found, and the range of motion of the metatarsophalangeal joints of amateur runners decreases as the LBS increases [32]. Shoes with higher LBS will limit the range of motion of the joint. Hoogkamer et al. discovered that high-LBS shoes decreased peak metatarsophalangeal joint dorsiflexion angles by 6° and 12° [13]. Stefanyshyn et al.’s research identified restricted activity in the plantarflexion/dorsiflexion of the metatarsophalangeal joint caused by heightened LBS in running shoes [33,34]. Not only that, shoes with a higher LBS increase the dorsiflexion of the metatarsophalangeal joint, while at the same time enhancing the foot’s ability to rotate outward as a potential compensatory strategy [35].

In terms of moment, this research found that the peak moments of knee flexion/extension, adduction/abduction, and ankle inversion/eversion were significantly different between S-1 and S-3 shoes in running shoes with different LBS transitions from level to uphill and that an increase in LBS led to a decrease in the peak moments at the knee and ankle joints. Zhou and Debelle’s research demonstrated an increased moment at the ankle joint and improved strength of the peroneus longus and peroneus brevis muscles when runners wore unstable shoes [36,37]. Amateur runners experience decreased ankle stabilization due to the inadequate support provided by low-stiffness running shoes. To ensure ankle stabilization while running, the muscles responsible for ankle motion must generate greater forces in the frontal plane to meet the demands for ankle stabilization. Wearing a running shoe with low stiffness has been shown to result in higher peak moments in ankle inversion and eversion. Willwacher also demonstrated a decrease in the mean ankle moment when running in shoes with medium stiffness compared to lower stiffness shoes [38]. Therefore, there was a higher possibility of injury when running in low-stiffness shoes. Researchers have usually assumed that there would be an increase in peak moments in the plantarflexion/dorsiflexion of the ankle. Only Roy and Stefanyshyn, however, have shown that there is an increase in the peak ankle moment as the LBS increases while jogging at a constant speed on a treadmill inclined at 1% [39]. Cigoja’s research demonstrated that wearing high-LBS shoes led to the metatarsophalangeal joints transitioning into the plantarflexion phase sooner. In amateur runners, the decrease in negative work generation at the time of metatarsophalangeal joints was accompanied by a corresponding increase in positive work, although this change did not significantly change the moment reduction [40]. This is consistent with our experimental results.

Hoogkamer’s research also found an increase in positive metatarsophalangeal joint plantarflexion/dorsiflexion work during the stance phase of running as the LBS of the running shoe increased [13]. From an energy loss perspective, increasing the LBS of a shoe stiffens the shoe, limiting the dorsiflexion of the metatarsophalangeal joints, slowing angular velocity, and decreasing energy loss at the metatarsophalangeal joints [41]. Therefore, wearing higher LBS running shoes can increase the exercise performance of amateur runners. In addition, the research found that, as the LBS increased, peak positive power and positive work of the knee in adduction/abduction decreased, but the positive work of the ankle in plantarflexion/dorsiflexion increased. Cigoja found that as LBS increased, there was a decrease in positive work at the knee joint. Additionally, the distribution of positive work shifts from the knee joint to the ankle joint in the experiment [34]. The muscle tendons at the proximal joints have lower energy storage and return capabilities compared to those at the distal joints, resulting in higher energy expenditure to produce the same amount of work [42,43]. Therefore, reallocating the workload among the lower limb joints benefits amateur runners in terms of enhancing their performance. Furthermore, the research found significant differences in ankle plantarflexion/dorsiflexion as well as knee flexion and extension negative work. In uphill running, negative work was mainly absorbed by the knee [44]. Willwacher found that the amount of negative work absorbed by the knee and ankle increased as the LBS increased when an amateur runner ran uphill with three different LBS shoes [45]. Thus, greater energy absorption is required at the knee joint to provide cushioning during the stance phase while running in high-LBS running shoes.

In summary, in this experiment, the biomechanical data of the lower limbs of amateur runners wearing three pairs of running shoes with different longitudinal bending stiffness were compared, and the shoe with the highest longitudinal bending stiffness in the sole was more suitable for amateur runners transitioning from level to uphill running. Increasing the LBS of running shoes can increase the running performance of amateur runners, promote safety, and decrease the risk of joint problems. However, excessive LBS in running shoes is not ideal for amateur runners. If the longitudinal bending stiffness of shoes is excessive, amateur runners may be hindered in terms of exercise performance [46]. Selecting the appropriate LBS running shoe based on amateur runner conditions and the level of running intensity was crucial to prevent avoidable injuries. 

## 5. Conclusions

Enhancing the LBS of running shoes can impact the biomechanics of the lower limb when transitioning from level to uphill running. The phenomenon of positive work shifting from proximal to distal joints in the lower extremity joints is observed, which would be beneficial in improving the efficient work performed in the lower limb. Amateur runners must absorb additional energy for cushioning in the knee joint when wearing high-LBS running shoes, which raises the risk of knee injury. In high-LBS running shoes, the metatarsophalangeal joint does more positive work and reduces negative work absorption. The angle of activity of the joints was reduced when wearing high longitudinal bending stiffness running shoes, and amateur runners were able to better control the stability of their joints. Therefore, high longitudinal bending stiffness running shoes were more suitable for amateur athletes to transition from level to uphill running. Furthermore, amateur runners could use these research findings to receive valuable suggestions when choosing running shoes with optimal LBS.

## Figures and Tables

**Table 1:** Motion range, peak moment, peak positive, and negative power and positive and negative work of knee, ankle, and thigh joints in three running shoe conditions.

Index	Joint Motion		S-1	S-2	S-3	F	*p*
Motion angle (°)	Knee	Flexion/Extension	28.05 ± 5.41	28.59 ± 4.86	25.48 ± 5.41	2.259	0.112
		Adduction/Abduction	4.34 ± 1.24 ^b^	4.92 ± 1.25 ^c^	3.57 ± 0.93 ^c^	7.779	0.001
		Intorsion/Extorsion	14.34 ± 6.69	14.25 ± 7.37	13.58 ± 7.34	0.075	0.927
	Ankle	Plantarflexion/Dorsiflexion	31.68 ± 8.60	32.50 ± 9.15	29.65 ± 9.14	0.677	0.511
		Inversion/Eversion	17.52 ± 4.04	18.24 ± 4.70	16.89 ± 4.33	0.556	0.576
	MTP	Plantarflexion/Dorsiflexion	13.03 ± 4.25 ^ab^	11.84 ± 3.30 ^ac^	8.56 ± 1.48 ^bc^	11.156	0.001
Peak moment (Nm/kg)	Knee	Flexion/Extension	3.51 ± 0.99 ^b^	3.33 ± 1.17 ^c^	2.24 ± 0.56 ^bc^	8.632	0.001
		Adduction/Abduction	−2.59 ± 1.21 ^ab^	−1.43 ± 0.31 ^a^	−1.85 ± 0.94 ^b^	7.362	0.001
		Intorsion/Extorsion	−1.05 ± 0.57	−0.86 ± 0.86	−0.59 ± 0.26	2.604	0.082
	Ankle	Plantarflexion/Dorsiflexion	−3.22 ± 1.81	−3.46 ± 0.64	−2.79 ± 0.66	1.901	0.158
		Inversion/Eversion	2.16 ± 1.04 ^b^	1.72 ± 0.68	1.52 ± 0.34 ^b^	4.1	0.021
	MTP	Plantarflexion/Dorsiflexion	0.56 ± 0.13	0.55 ± 0.18	0.51 ± 0.15	0.44	0.646
Peak positive power (W/kg)	Knee	Flexion/Extension	1.68 ± 0.33 ^ab^	1.37 ± 0.22 ^a^	1.17 ± 0.20 ^b^	8.868	0.001
		Adduction/Abduction	1.92 ± 0.70 ^ab^	1.37 ± 0.52 ^a^	1.26 ± 0.32 ^b^	5.608	0.007
		Intorsion/Extorsion	0.15 ± 0.09	0.13 ± 0.11	0.11 ± 0.15	0.668	0.517
	Ankle	Plantarflexion/Dorsiflexion	6.89 ± 0.70 ^b^	6.20 ± 1.01 ^c^	4.79 ± 0.85 ^bc^	13.92	0.001
		Inversion/Eversion	0.54 ± 0.11	0.52 ± 0.15	0.49 ± 0.12	0.355	0.704
	MTP	Plantarflexion/Dorsiflexion	1.65 ± 0.74	2.04 ± 0.96	2.16 ± 0.85	1.579	0.217
Peak negative power (W/kg)	Knee	Flexion/Extension	−2.14 ± 0.59	−2.20 ± 0.38	−2.13 ± 0.4	0.071	0.932
		Adduction/Abduction	−1.76 ± 0.50 ^ab^	−0.76 ± 0.55 ^a^	−1.17 ± 0.70 ^b^	9.562	0.001
		Intorsion/Extorsion	−0.22 ± 0.25	−0.13 ± 0.07	−0.14 ± 0.06	1.575	0.217
	Ankle	Plantarflexion/Dorsiflexion	−4.30 ± 0.93 ^ab^	−3.36 ± 0.74 ^a^	−3.07 ± 0.58 ^b^	6.284	0.006
		Inversion/Eversion	−0.44 ± 0.12	−0.45 ± 0.35	−0.46 ± 0.11	0.014	0.986
	MTP	Plantarflexion/Dorsiflexion	−1.80 ± 0.89	−1.52 ± 0.61	−1.79 ± 0.85	0.656	0.524
Positive work (J/kg)	Knee	Flexion/Extension	0.24 ± 0.13	0.32 ± 0.12	0.29 ± 0.08	2.387	0.102
		Adduction/Abduction	0.12 ± 0.09 ^b^	0.16 ± 0.09 ^c^	0.06 ± 0.01 ^bc^	9.469	0.001
		Intorsion/Extorsion	0.09 ± 0.08	0.06 ± 0.06	0.07 ± 0.10	0.879	0.42
	Ankle	Plantarflexion/Dorsiflexion	0.54 ± 0.36	0.41 ± 0.09 ^c^	0.64 ± 0.23 ^c^	3.409	0.041
		Inversion/Eversion	0.31 ± 0.16 ^a^	0.20 ± 0.07 ^a^	0.23 ± 0.07	4.598	0.015
	MTP	Plantarflexion/Dorsiflexion	0.05 ± 0.02 ^b^	0.07 ± 0.05	0.09 ± 0.04 ^b^	5.005	0.009
Negative work (J/kg)	Knee	Flexion/Extension	−0.24 ± 0.13 ^b^	−0.34 ± 0.15	−0.42 ± 0.11 ^b^	7.848	0.001
		Adduction/Abduction	−0.13 ± 0.10	−0.09 ± 0.09	−0.12 ± 0.19	0.491	0.614
		Intorsion/Extorsion	−0.07 ± 0.06 ^b^	−0.09 ± 0.02 ^c^	−0.03 ± 0.02 ^bc^	14.783	0.001
	Ankle	Plantarflexion/Dorsiflexion	−0.28 ± 0.23	−0.21 ± 0.05 ^c^	−0.37 ± 0.10 ^c^	4.726	0.013
		Inversion/Eversion	−0.28 ± 0.13	−0.25 ± 0.17	−0.17 ± 0.06	2.881	0.065
	MTP	Plantarflexion/Dorsiflexion	−0.09 ± 0.08	−0.07 ± 0.07	−0.06 ± 0.04	1.283	0.283

Notes: (a) represents a statistically significant difference between S-1 shoe and S-2 shoe data; (b) represents a statistically significant difference between S-1 shoe and S-3 shoe data; (c) represents a statistically significant difference between S-2 shoe and S-3 shoe data.

## Data Availability

The data that support the findings of this study are available on reasonable request from the corresponding author. The data are not publicly available due to privacy or ethical restrictions.
